# Sleep health composites are associated with the risk of heart disease across sex and race

**DOI:** 10.1038/s41598-022-05203-0

**Published:** 2022-02-07

**Authors:** Soomi Lee, Christina X. Mu, Meredith L. Wallace, Ross Andel, David M. Almeida, Orfeu M. Buxton, Sanjay R. Patel

**Affiliations:** 1 School of Aging Studies, University of South Florida, 4202 E. Fowler Avenue, MHC 1344, Tampa, FL 33620 USA; 2 Department of Psychiatry, Statistics and Biostatistics, University of Pittsburgh, Pittsburgh, PA USA; 3 Department of Neurology, Second Faculty of Medicine, Charles University/Motol University Hospital, Prague, Czech Republic; 4 Department of Human Development and Family Studies, Pennsylvania State University, University Park, PA USA; 5 Department of Biobehavioral Health, Pennsylvania State University, University Park, PA USA; 6 Department of Medicine, University of Pittsburgh, Pittsburgh, PA USA

**Keywords:** Cardiovascular diseases, Risk factors

## Abstract

We examined whether subjectively and objectively measured sleep health composites have a relationship with heart disease. 6,820 adults (*M*_*age*_ = 53.4 years) from the Midlife in the United States study provided self-reported sleep characteristics and heart disease history. A smaller sample (*n* = 663) provided actigraphy sleep data. We tested two sleep health composites, based on self-report only and both self-report and actigraphy, across multiple sleep dimensions. We used a weighted sum approach, where higher scores indicated more sleep health problems. Modified Poisson regressions adjusted for sociodemographics and known risk factors. Having more sleep health problems was associated with a higher risk of heart disease using the self-report sleep health composite (a*RR* = 54%, *P* < .001) and the actigraphy/self-report composite (a*RR* = 141%, *P* < .001). Individual sleep dimensions of satisfaction, alertness, and efficiency (from the self-report composite) and regularity, satisfaction, and timing (from the actigraphy/self-report composite) were associated with the risk of heart disease. The effect size of each sleep health composite was larger than the individual sleep dimensions. Race moderated the association between the actigraphy/self-report sleep health composite and heart disease. There was no significant moderation by sex. Findings suggest poorer sleep health across multiple dimensions may contribute to heart disease risk among middle-aged adults.

## Introduction

Insufficient or poor sleep is a significant risk factor for heart disease^1–5^. Studies have mostly used single sleep measures (often focusing only on sleep duration, quality, or insomnia). However, a composite of multidimensional sleep health may be more predictive of heart disease than single sleep measures^3,6–8^. For example, an individual who has any two sleep problems simultaneously (e.g., shorter sleep duration and unsatisfactory sleep) may have a higher risk of heart disease than an individual who has only one sleep problem (e.g., shorter sleep duration alone). Examining the degree of multidimensional sleep health and its association with the risk of heart disease is important in research on middle-aged adults, as multiple sleep problems may be prevalent in this population^8^. Poor sleep health in middle-aged adults may lead to the loss of productivity^9,10^, impaired immune functioning^11^, increased risks of heart disease^4^, other illnesses in later life, and early mortality^7,12,13^. As sleep health is modifiable^14,15^, understanding multidimensional sleep health in middle adulthood may contribute to future prevention strategies aimed to mitigate the risk of heart disease, which is a leading cause of death in the United States^16^.

Sleep health is multifaceted and complex^6,8,17^. Buysse^18^ suggests key dimensions to define and measure multidimensional sleep health: Regularity in sleep, Satisfaction with sleep, Alertness during waking hours, Timing of sleep, Sleep Efficiency, and Sleep Duration (Ru-SATED). There is an emerging paradigm shift towards studying multidimensional sleep, instead of single indicators, and its associations with health outcomes. For example, a sleep health composite (based on self-report) is associated with multiple health outcomes in adolescents, such that poorer sleep health is related to higher depressive and anxiety symptoms, more social problems related to friends and family, and higher odds of obesity^19^. A composite of poorer sleep health (based on self-report only) is also associated with higher odds of depression symptoms in older women^20^; and poorer sleep health (based on both self-report and actigraphy) is predictive of mortality in older men^21^. Furthermore, a sleep health composite (based on both self-report and actigraphy) in middle adulthood has been studied in relation to cardiometabolic outcomes, such that poorer sleep health is associated with higher odds of hypertension and diabetes^6^.

Yet, more research during middle adulthood is needed to understand the links between multidimensional sleep health and specific pathophysiological outcomes such as heart disease. Middle adulthood spans for a longer time of life and consists of diverse (and more stressful) life experiences across work and family^22^. Additionally, middle adulthood is when atherosclerosis (a precursor of heart disease) develops and age-related sleep issues begin to arise^23^. Thus, middle adulthood may involve more and diverse sleep health issues, which can be better captured by a sleep health composite rather than by single measures of sleep^8,24^. Examining multidimensional sleep health in middle adulthood and its associations with the risk of heart disease may increase our understanding on the importance of maintaining good sleep health during middle adulthood for reducing the risk of a leading cause of death. Potentially diverse sleep health issues in middle adulthood also raises a question whether the predictive property of sleep health may depend on specific sleep measures used. Indeed, previous research has used different sleep measures to understand “sleep health” (e.g.^25^), making it difficult to provide a consistent guideline for research and practical settings. To address these gaps, we operationalize two different sleep health composites in middle-aged adults and link them to the risk of physician-diagnosed heart disease. If results are consistent between two measures, it may suggest that, rather than specific sleep measures, the combination of multiple sleep measures is more important for the risk of heart disease.

Another important thing to consider is differences by sex and race. Sex and race appear to play roles in sleep quality, sleep duration, and heart disease risk. Previous studies regarding sex differences in sleep and heart disease are inconsistent and findings differ by specific sleep dimensions. For example, men are more likely to have an earlier onset of obstructive sleep apnea, and may be at a 7–8% higher risk of heart disease compared to women^17,26^. However, women are more likely to report insomnia symptoms due to sex hormone differences affecting circadian rhythms^27^. Regarding differences by race, Black individuals are more likely to have shorter sleep duration (< 6 h per night)^28,29^, more sleep disturbances^30^, and overall worse global sleep scores compared to White counterparts^31^. Together, these studies suggest that it is important to consider potential differences by sex and race in the sleep health and heart disease relationship.

The current study examined the associations of multidimensional sleep health with the risk of heart disease in middle-aged adults. To assess “how many” sleep health problems co-occur within an average adult (e.g., irregular sleep timing coupled with less than 6 h of sleep), we used two independent sleep health composites–one based on self-report responses only and the other one based on both actigraphy and self-report. The two sleep health composites used different sleep variables but captured common sleep health dimensions suggested by Buysse^18^. We hypothesized that more sleep health problems would be associated with a higher risk of heart disease. Additionally, we tested potential differences by sex and race in sleep health, heart disease, and their interrelationships. We expected that women would have more sleep health problems (especially based on self-report) and men would have a higher risk of heart disease. We also expected that racial minorities would have more sleep health problems and a higher risk of heart disease than White individuals. In terms of differences by sex and race in the sleep health and heart disease relationship, we did not formulate specific hypotheses due to inconsistent findings in the literature. Our approach lends itself to characterizing a “sleep health” message that will be more effective in motivating the public to engage in multiple sleep health behaviors that may have synergistic effects on decreasing the risk of heart disease.

## Methods

### Participants and procedures

**Figure 1:**
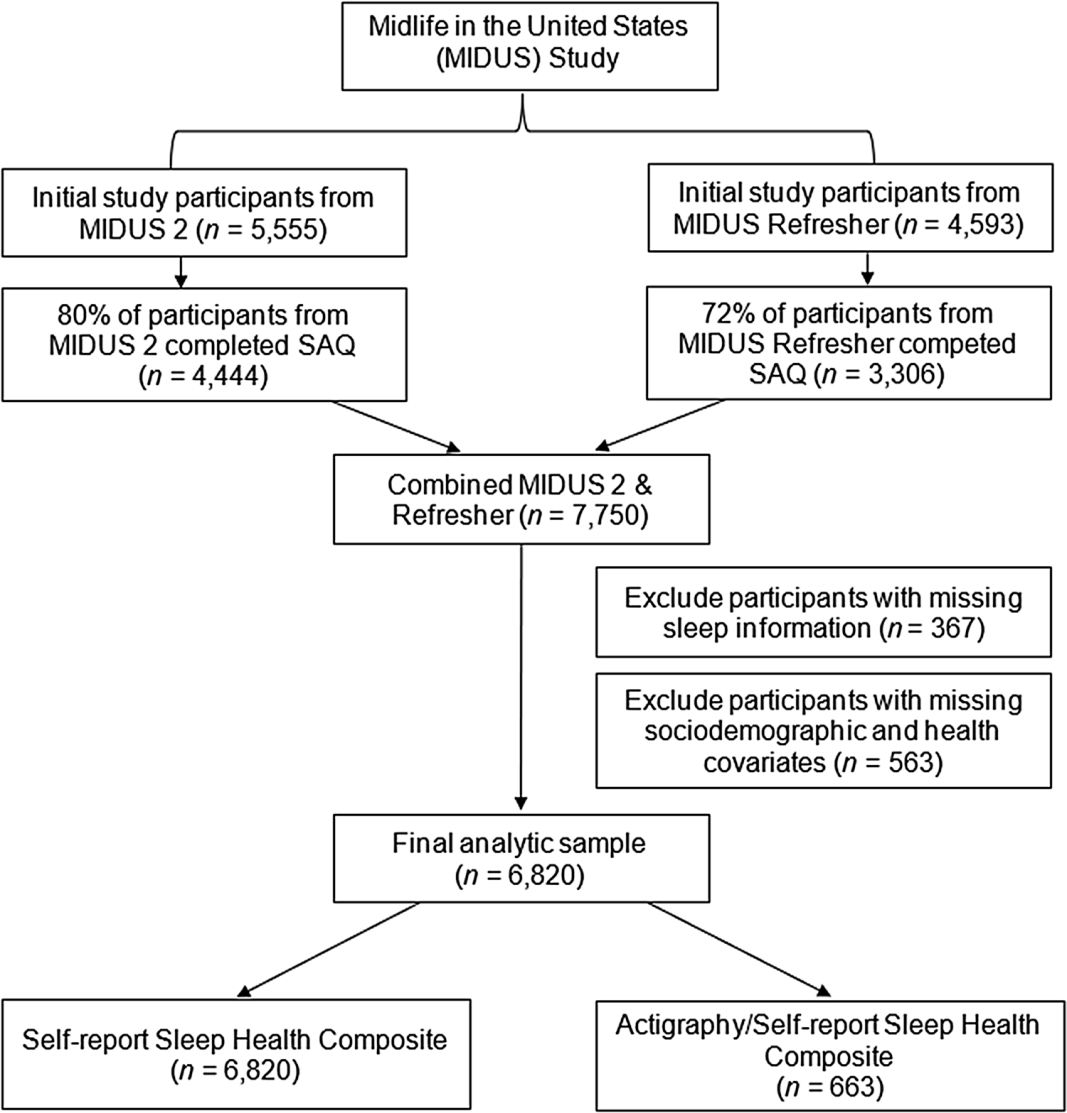
Flowchart of MIDUS analytic sample; SAQ = Self-Administered Questionnaire.

Data came from the Midlife in the United States (MIDUS) study. Comprehensive assessments of sleep measures, including self-administered questionnaires and sleep actigraphy, were captured from the second-wave sample of the study recruited between 2004–2009 (M2; *N* = 5,555) and from the Refresher sample recruited between 2011–2014 (MR; *N* = 4,593). A subset of participants from M2 and MR were invited to wear a sleep Actiwatch and complete a daily sleep diary for seven consecutive days to measure objective and subjective assessments of sleep (referred to as the Biomarker Project). Comprehensive study details can be found elsewhere^32–35^. Figure 1 displays the sample flowchart. Our final analytic samples were 6,820 adults who provided self-report sleep data and 663 adults who provided both self-report and actigraphy sleep data. The MIDUS study was approved by all appropriate Institutional Review Boards, and all MIDUS participants provided informed consent. The current study was exempt from the IRB review process because it used publicly available, de-identifiable data. All methods were performed in accordance with the relevant guidelines and regulations.

### Measures

#### Sleep health composites

Guided by the Ru-SATED model, we created two sleep health composite variables to capture Regularity, Satisfaction, Alertness, Timing, Efficiency, and Duration^18^–one composite was captured using self-reported sleep variables and the other using both actigraphy and self-report sleep variables. The *self-report sleep health composite* captured five of the six Ru-SATED dimensions (not sleep timing), and *the actigraphy/self-report sleep health composite* captured all six dimensions in the Ru-SATED model (see Table 1).

**Table 1:** Sleep health dimensions to construct two sleep health composites.

Dimension	Self-report sleep health composite				Actigraphy/self-report sleep health composite			
	Variable	Assessment / Item	*Range*	*M* (*SD*)	Variable	Assessment / Item	*Range*	*M* (*SD*)
Regularity^8,37^	Sleep Debt	Difference between workday sleep duration and non-workday sleep duration	− 13 to 12 min	0.58 (1.12)	Irregularity of Sleep Midpoint	Standard deviation (SD) of sleep midpoint	0 to 9.97 SDs	0.84 (0.83)
Satisfaction^8,25,44^	Trouble Falling Asleep	Have trouble falling asleep	1: Sometimes, Often, or Almost Always; 0: Rarely or Never Variables Summed: 0–4	1.84 (1.45)	Pittsburgh Sleep Quality Index (PSQI)	During the past month, how would you rate your sleep quality overall?	0 (Very Good) to 3 (Very Bad)	1.01 (0.68)
	Nocturnal Awakenings	Wake up during the night and have difficulty going back to sleep						
	Early Awakenings	Wake up too early in the morning and be unable to get back to sleep						
	Unrested Upon Waking	Feel unrested during the day, no matter how many hours of sleep you had						
Alertness ^38^	Nap Frequency	During a usual week, how many times do you nap for 5 min or more?	0 to 13 times	2.01 (2.52)	Alertness (Diary)	“How alert were you today?”	1 (Most Alert) to 5 (Not Alert at All)	2.04 (0.74)
Timing^18^	Not captured through survey				Sleep Midpoint	Midpoint from bedtime to waketime	− 4.05 to 14.21	3.00 (1.40)
Efficiency^6^	Sleep Onset Latency	How long does it usually take you to fall asleep at bedtime?	0 to 4 h	0.49 (0.55)	Sleep Efficiency*	% of Time Asleep Between Bedtime and Waketime	33.84 to 94.99%	79.54 (10.70)
Duration^8^	Workday Sleep Duration*	How much sleep do you usually get at night (or in your main sleep period) on weekdays or workdays?	0 to 13 h	6.90 (1.28)	Sleep Time*	Time in bed – Sleep onset latency –Wake After Sleep Onset (WASO)	2.00 to 10.27 h	6.17 (1.11)

*Indicates that continuous variables were reverse coded, so that higher values represent poorer sleep.

The use of these two different composites was to see whether the proposed relationship between multidimensional sleep health and heart disease would be replicated across different measures assessing sleep health. In the two sleep health composites, each dimension was measured using different sleep measures, depending on the relevance and data availability. For example, the self-report regularity dimension was captured by sleep debt, which refers to a significant gap between weekday sleep and weekend sleep^36^. Sleep debt is closely related to jet lag or circadian rhythm misalignment^37^, which refers to irregularity in sleep behavior. For the self-report alertness dimension, we assessed nap frequency as it was the only available survey measure related to lack of alertness. Frequent naps (> 2 naps per week) have been found to be associated with incident cardiovascular events^38^.

Each of the composites was created using a weighted sum. This approach is optimized for accurate prediction because individual sleep dimensions may be differentially associated with the risk of heart disease. We first ran unadjusted models regressing the risk of heart disease on all the sleep health dimensions simultaneously. We used z-scores of the sleep dimensions in order to allow for comparability across sleep dimensions measured on different scales. Next, we extracted the beta coefficients from this model and inputted them into an equation where all indicators were summed based on their weights (see Table 2). Higher weighted values indicated that the sleep dimension was more important for determining the risk of heart disease. We also conducted a sensitivity check with unweighted composites which assume all sleep health features contribute equally based on pre-established cut points (see Table 3 for how we created these measures).

**Table 2:** Results from unadjusted models with individual sleep health dimensions and the risk of heart disease.

Variable	*B*	SE	*p*	95% CI
**Model 1. self-report model**				
Regularity^1^	− 0.08	0.03	.004	− 0.14, − 0.03
Satisfaction	0.14	0.03	<.001	0.08, 0.20
Alertness	0.19	0.02	<.001	0.14, 0.23
Efficiency	0.10	0.02	<.001	0.06, 0.15
Duration	− 0.06	0.03	.058	− 0.12, 0.002
**Model 2. actigraphy/self-report model**				
Regularity^1^	0.03	0.09	.779	− 0.15, 0.21
Satisfaction	0.17	0.09	.045	0.004, 0.34
Alertness	0.04	0.10	.693	− 0.16, 0.23
Timing	0.22	0.06	<.001	0.11, 0.33
Efficiency	0.10	0.11	.360	− 0.11, 0.31
Duration	− 0.02	0.13	.862	− 0.28, 0.23

^1^For each sleep health dimension, higher scores indicated poorer sleep (i.e., irregularity, poorer satisfaction, lack of alertness, inefficiency, later midpoint timing, and shorter duration). All sleep dimensions were treated continuously and z-scored, then entered simultaneously in the same model. The beta coefficients from this table were used to create the weighted-regression sleep health composites:

**Self-Report Weighted Sleep Health Composite** $=(-0.08*\mathrm{Regularity})+(0.14*\mathrm{Satisfaction})+(0.19*\mathrm{Alertness})+(0.10*\mathrm{Efficiency})+(-0.06*\mathrm{Duration})$.

**Actigraphy/self-report Weighted Sleep Heath Composite** $=(0.03*\mathrm{Regularity})+(0.17*\mathrm{Satisfaction})+(0.04*\mathrm{Alertness})+\left(0.22*\mathrm{Timing}\right)+(0.10*\mathrm{Efficiency})+(-0.02*\mathrm{Duration})$.

**Table 3:** Cut points used to construct unweighted sleep health composites.

Dimension	Self-report sleep health composite		Actigraphy/self-report sleep health composite	
	Variable	Cut point	Variable	Cut point
Regularity^8,37^	Sleep Debt	1: Absolute value > 60 min 0: Absolute value ≤ 60 min	Irregularity (SD) of Sleep Midpoint	1: > Mean + 1SD (1.64) 0: ≤ Mean + 1SD (1.64)
Satisfaction^8,25,44^	Trouble Falling Asleep	1: “Sometimes, Often, or Almost Always” on at least 1 of 4 items 0: “Rarely or Never” for all 4 items	Pittsburgh Sleep Quality Index (PSQI)	0 (Very Good) to 3 (Very Bad) 1: ≥ 2 0: < 2
	Nocturnal Awakenings			
	Early Awakenings			
	Unrested Upon Waking			
Alertness^38^	Nap Frequency	1: > 2 nap per week 0: ≤ 2 nap per week	Alertness (Diary)	5 (Not Alert at All) to 1 (Most Alert) 1: > 3 0: ≤ 3
Timing^18^	Not captured through survey		Sleep Midpoint	1: Early (≤ 2 AM) or Late (> 4 AM) 0: ≤ Middle (> 2 & ≤ 4 AM)
Efficiency^6^	Sleep Onset Latency	1: > 30 min 0: ≤ 30 min	Sleep Efficiency	0%-100% 1: < 85% 0: ≥ 85%
Duration^8^	Workday Sleep Duration	1: < 6 or > 8 h 0: ≥ 6 & ≤ 8 h	Sleep Time	1: < 6 or > 8 h 0: ≥ 6 & ≤ 8 h

Cut points were determined using empirical evidence from previous research. The satisfaction dimension was assessed by four items related to insomnia symptoms and feeling unrested upon waking, which were highly loaded to sleep quality factor in the Sleep Health Index developed by the National Sleep Foundation^25^. Binary indicators were summed, so that possible scores ranged from 0–5 for the self-report sleep health composite and 0–6 for the actigraphy/self-report sleep health composite. Higher numbers indicated more sleep health problems.

#### Diagnoses of heart disease

Participants were asked, “Have you ever had heart trouble suspected or confirmed by a doctor?” and “Have you ever had a severe pain across the front of your chest lasting half an hour or more?” If participants responded “yes” to the first question, then a follow-up question asked, “What was the diagnosis?” If participants responded “yes” to the second question then a follow-up question asked, “Which did the doctor say it was?” For both follow-up questions, the lists of possible diagnoses were consistent and included: (1) heart attack; (2) angina; (3) high blood pressure; (4) valve disease, mitrovalve prolapse, aortic insufficiency, bicuspid aortic valve; (5) hole in heart, atrial septal defect, ventricular septal defect; (6) blocked/closed artery, coronary artery disease, coronary heart disease, ischemia; (7) irregular/fast heartbeat, arrhythmia; heart murmur; (8) heart failure, congestive heart failure, enlarged heart; and/or (9) other. We excluded high blood pressure as it is a risk factor of heart disease rather than a heart disease condition^39^. “Yes” responses to any of the heart disease diagnoses were coded as having a diagnosis of heart disease (= 1 vs. 0 = no heart disease).

#### Covariates

We controlled for sociodemographics and known heart disease risk factors including age, sex, race/ethnicity, education, work status, BMI, diabetes, hypertension, smoking status, depression, anxiety, and the month data collection occurred since a previous study found that sleep complaints varied across seasons^40^. Depression and anxiety were measured using the World Mental Health Organization’s Composite International Diagnostic Interview Short Form^41^. In the depression and anxiety questionnaires, sleep-related items were removed. Additional analyses were conducted controlling for family history of heart disease and physical activity; both of which were only available in the Biomarker Project. Family history of heart disease was assessed by asking participants, “Has anyone in your immediate family (blood relative only) had heart disease?” Physical activity was assessed by asking participants, “Do you engage in regular exercise, or activity, of any type for 20 min or more at least 3 times/week?” Response options for both questions were coded, 0 = No and 1 = Yes.

### Statistical analyses

We used modified Poisson regression models with robust variance estimation in SAS v9.4 procedure GENMOD to test the associations of each sleep health composite with the risk of heart disease. This method allows for estimation of relative risks (*RR*; adjusted relative risks: a*RR*) and is recommended when the prevalence of outcome is > 10%^42,43^. The self-report only and actigraphy/self-report sleep health composites were included in separate models. To test differences by sex (men vs. women) and race (non-Hispanic Black, all other races, vs. non-Hispanic White), we used chi-squared tests, *t*-tests, and interaction terms between sleep health composites and each of sex and race in separate models. Continuous covariates were z-scored to improve interpretability in relation to each sleep health composite (i.e., weighted sum of z-scores). Significance was determined by using two-tailed test with a *p*-value of <.05.

## Results

Sample characteristics and descriptive statistics are shown in Table 4. The correlations among all individual sleep variables ranged from ± 0.01 to 0.62, meaning that they were related but had unique variances. The correlation between the two sleep health composites indicated that they were moderately related (*r* = 0.35, *P* <.001).

**Table 4:** MIDUS sample characteristics and descriptive statistics.

	Self-report Only Sample (*n* = 6,820)	Actigraphy/Self-report Sample (*n* = 663)	Difference Test	
	*Mean* (*SD*) or *n* (%)			*p*
**Sociodemographics and health covariates**				
Age	53.45 (13.27)	52.48 (12.31)	1.92	.055
**Sex**			4.83	.028
Female	3698 (54.2%)	389 (58.7%)		
Male	3122 (45.8%)	274 (41.3%)		
**Race**			51.99	<.001
Non-Hispanic White	5193 (76.1%)	443 (66.8%)		
Non-Hispanic Black	1118 (16.4%)	182 (27.5%)		
**All other races**				
Native American or Alaska Native, Aleutian Islander, Eskimo	96 (1.4%)	12 (1.8%)		
Asian	49 (0.7%)	6 (0.9%)		
Native Hawaiian or Pacific Islander	7 (0.1%)	0 (0.0%)		
Other/Other (Specify)	148 (2.2%)	16 (2.4%)		
Hispanic	209 (3.1%)	4 (0.6%)		
**Education**			4.82	.185
No High School Diploma or GED	434 (6.4%)	46 (6.9%)		
High School Diploma or GED	1642 (24.1%)	135 (20.4%)		
Some College or Associates Degree	2021 (29.6%)	201 (30.3%)		
Bachelor's Degree or Higher	2723 (39.9%)	281 (42.4%)		
Employed/Self-employed	3718 (54.5%)	371 (56.0%)	0.51	.477
BMI (kg/m^2^)	28.64 (6.57)	29.45 (6.83)	− 3.01	.003
Has Diabetes	737 (10.8%)	80 (12.1%)	0.99	.321
Has Hypertension	2658 (39.0%)	261 (39.4%)	0.04	.843
**Smoking**			2.17	.337
Never smoked	3671 (53.8%)	367 (55.4%)		
Former smoker	2108 (30.9%)	209 (31.5%)		
Current smoker	1041 (15.3%)	87 (13.1%)		
Depression (Range = 0–6: Higher)	0.56 (1.55)	0.55 (1.50)	0.16	.876
Anxiety (Range = 0–8: Higher)	0.12 (0.77)	0.15 (0.89)	− 0.75	.452
**Main variables**				
Self-report Sleep Health Composite (Range = − 1.31 to 1.61: Higher means poorer sleep health)	− 0.008 (0.29)	− 0.01 (0.29)	0.20	.844
Actigraphy/Self-report Sleep Health Composite (Range = − 0.90 to 2.17: Higher means poorer sleep health)	–	0.001 (0.37)	− 0.15	.878
Heart Disease	1196 (17.5%)	96 (14.5%)	2.12	.034

The self-report only sample had less females, more non-Hispanic White and less non-Hispanic Black individuals, lower BMI, and higher prevalence of heart disease compared to the actigraphy/self-report sample.

**Figure 2:**
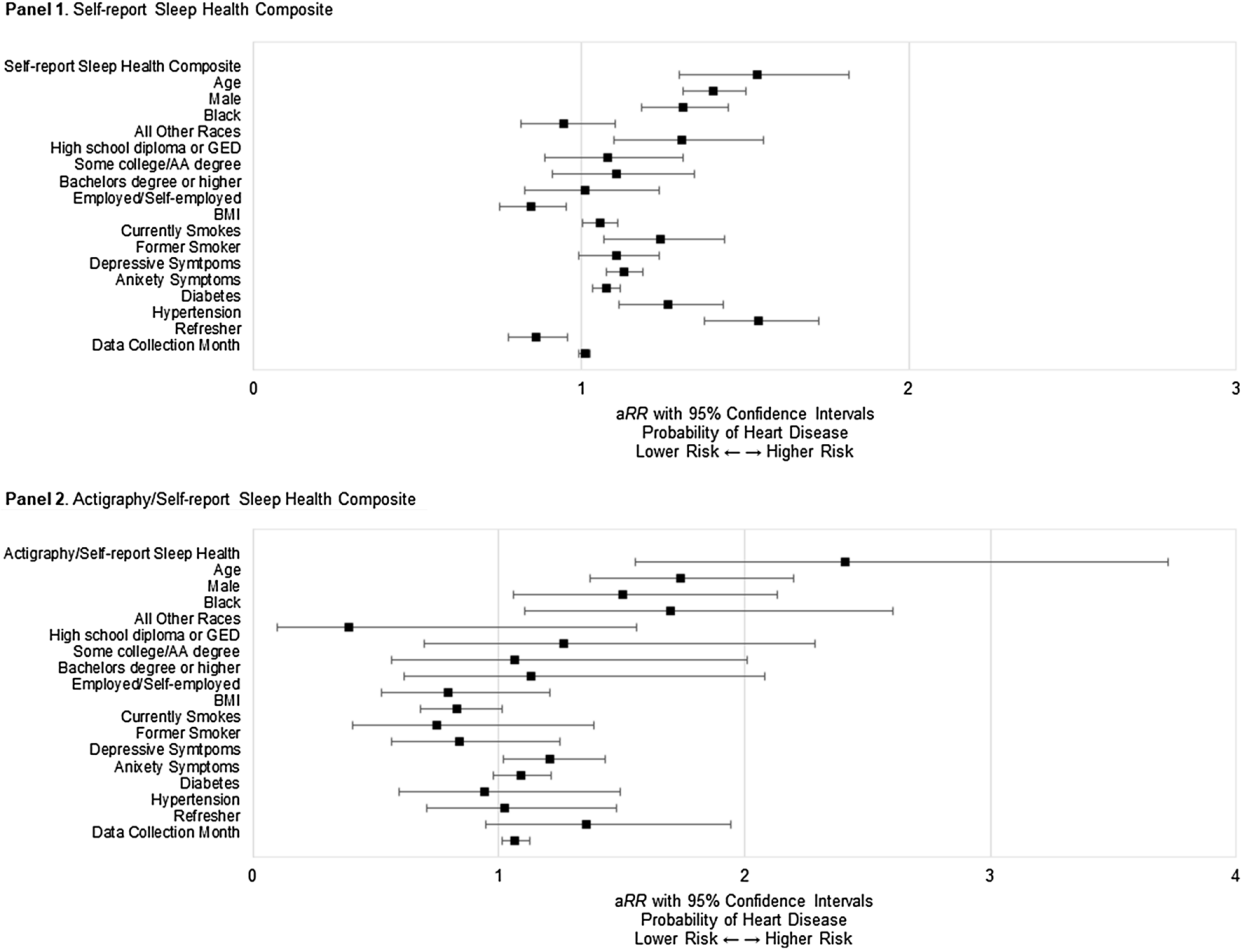
Results of modified Poisson analyses displaying the relative risk of heart disease by the weighted sleep health composites and covariates.

Results from a fully adjusted modified Poisson regression models showed a significant association of the self-report sleep health composite with the risk of heart disease. Each unit increase in poor sleep health was associated with 54% higher risk of heart disease (*B* = 0.43, *SE* = 0.09, 95% CI [0.26, 0.60], a*RR* = 1.54, *P* < .001) (Fig. 2, Panel 1). For the actigraphy/self-report sleep health composite, each unit increase in poor sleep health was associated with 141% higher risk of heart disease (*B* = 0.88, *SE* = 0.22, 95% CI [0.44, 1.32], a*RR* = 2.41, *P* < .001), after adjusting for all covariates (Fig. 2, Panel 2).

**Figure 3:**
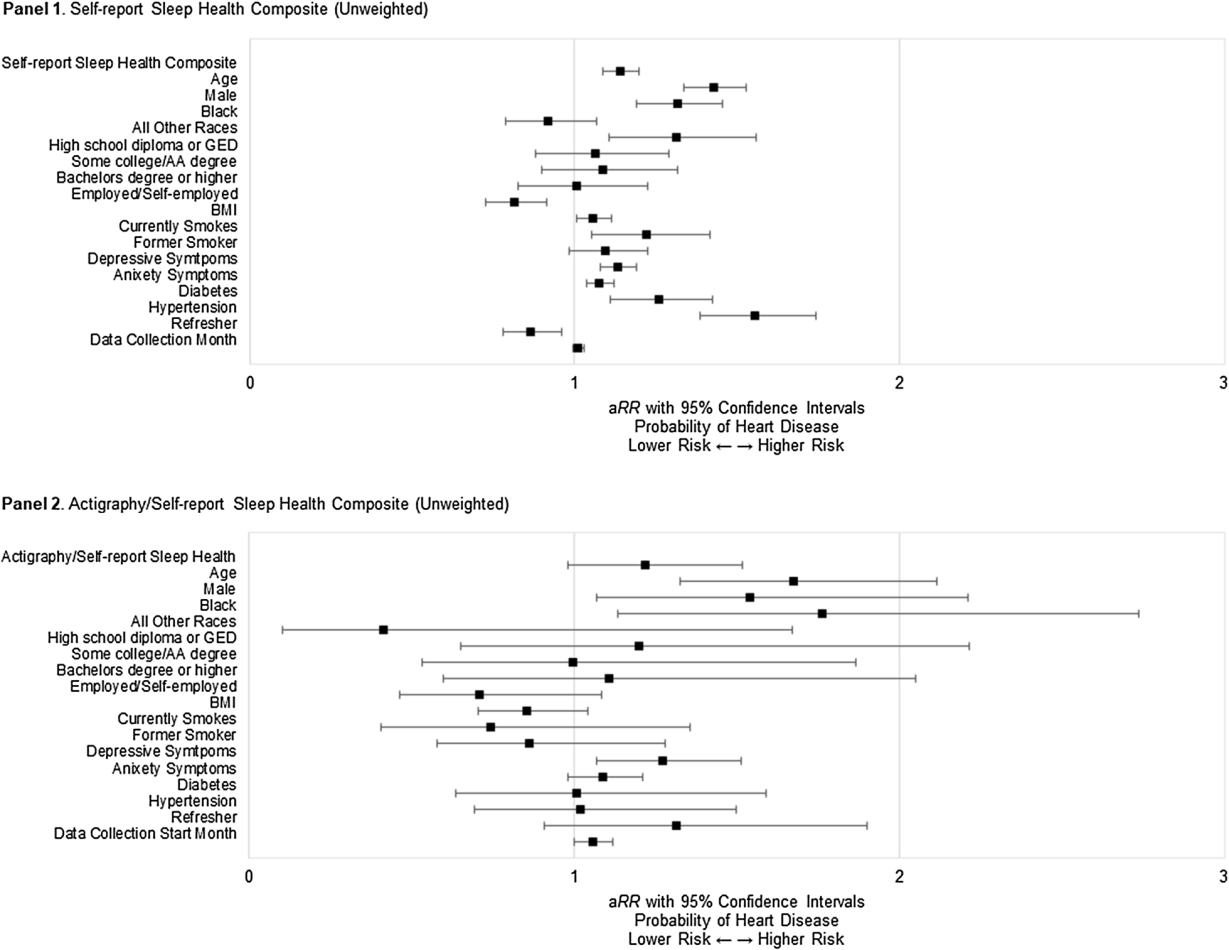
Results of modified Poisson analyses displaying the relative risk of heart disease by unweighted sleep health composites and covariates.

As a sensitivity check, we repeated the analyses using unweighted sleep health composites. Results were generally consistent but weaker. For the self-report unweighted composite, each additional dimension of poor sleep health was associated with 14% higher risk of heart disease (*B* = 0.13, *SE* = 0.03, 95% CI [0.08, 0.18], a*RR* = 1.14, *P* < .001) (Fig. 3, Panel 1). For the actigraphy/self-report unweighted composite, the association between poor sleep health and the risk of heart disease was in the expected direction but did not reach statistical significance (*B* = 0.20, *SE* = 0.11, 95% CI [− 0.02, 0.42], a*RR* = 1.22, *P* = .073) (Fig. 3, Panel 2).

### Differences by sex and race

There were significant differences in sleep health composites and heart disease by sex and race (Supplementary Fig. S1). Compared to men, women had slightly more sleep health problems based on the self-report (but no sex difference in the actigraphy/self-report sleep health composite). Men were more likely to have heart disease compared to women. However, sex did not moderate the association between sleep health composites and the risk of heart disease.

**Figure 4:**
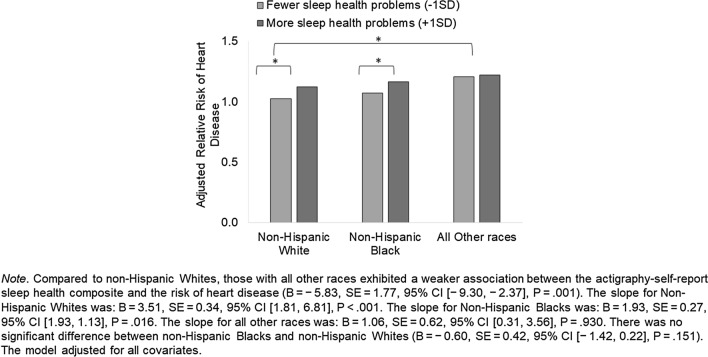
Moderation by race in the association between actigraphy/self-report sleep health composite and the risk of heart disease. *Note.* Compared to non-Hispanic Whites, those with all other races exhibited a weaker association between the actigraphy-self-report sleep health composite and the risk of heart disease (*B* = − 5.83, *SE* = 1.77, 95% CI [− 9.30, − 2.37], *P* = .001). The slope for Non-Hispanic Whites was: *B* = 3.51, *SE* = 0.34, 95% CI [1.81, 6.81], *P* < .001. The slope for Non-Hispanic Blacks was: *B* = 1.93, *SE* = 0.27, 95% CI [1.93, 1.13], *P* = .016. The slope for all other races was: *B* = 1.06, *SE* = 0.62, 95% CI [0.31, 3.56], *P* = .930. There was no significant difference between non-Hispanic Blacks and non-Hispanic Whites (*B* = − 0.60, *SE* = 0.42, 95% CI [− 1.42, 0.22], *P* = .151). The model adjusted for all covariates.

Turning to racial differences, non-Hispanic Black individuals had the highest number of sleep health problems, followed by all other races, and then non-Hispanic White individuals. This was observed in both the self-report and actigraphy/self-report sleep health composites. Race was significantly associated with heart disease, only in the actigraphy/self-report sample. Non-Hispanic Black individuals had the highest prevalence, followed by non-Hispanic White individuals, and other races. There was one significant moderation by race in the association between the actigraphy/self-report sleep health composite and heart disease. Compared to non-Hispanic White individuals, those with all other races exhibited a weaker association between the actigraphy-self-report sleep health composite and the risk of heart disease (*B* = − 5.83, *SE* = 1.77, 95% CI [− 9.30, − 2.37], *P* = .001). See Fig. 4 for the nature of this interaction and slope estimates. For non-Hispanic Whites, more sleep health problems were associated with significantly higher risk of heart disease. This was similar for non-Hispanic Blacks with no difference to non-Hispanic Whites (*B* = − 0.60, *SE* = 0.42, 95% CI [− 1.42, 0.22], *P* = .151). However, for those with other races, the slope was not significant. Race did not moderate the association between the self-report sleep health composite and the risk of heart disease. See Supplementary Table S1 for results from models stratified by sex or by race. Effect sizes of sleep health composites seemed to vary by groups.

### Supplemental analyses

All results were consistent with or without history of stroke in the heart disease outcome (results not shown, but available upon request). We also compared the effect sizes of individual sleep health dimensions in fully adjusted models (Supplementary Fig. S2) vs. the found effect of a sleep health composite. Among the self-report sleep health dimensions, satisfaction (a*RR* = 0.14, 95% CI [0.03, 0.09], *P* < .001), alertness (a*RR* = 0.05, 95% CI [0.004, 0.10], *P* = .033), and efficiency (a*RR* = 0.08, 95% CI [0.04, 0.13], *P* < .001) were independently associated with heart disease. Regularity and duration dimensions were not significantly associated with heart disease. For the actigraphy/self-report sleep health dimensions, regularity (a*RR* = 0.17, 95% CI [0.04, 0.30], *P* = .009), satisfaction (a*RR* = 0.20, 95% CI [0.03, 0.37], *P* = .024) and timing (a*RR* = 0.24, 95% CI [0.11, 0.36], *P* < .001) dimensions were independently associated with heart disease. Overall, the effect sizes of the significant individual dimensions were smaller than that of the sleep health composite.

Next, we repeated our analysis with the self-report sleep health composite in the smaller sample that provided both actigraphy and self-report sleep data. The previously significant effect of self-report sleep health composite became non-significant (*B* = 0.14, *SE* = 0.09, 95% CI [− 0.04, 0.32], a*RR* = 1.15, *P* = .137). Lastly, we compared results before and after controlling for family history of heart disease and physical activity. Results were unchanged with the self-report composite (*B* = 0.40, *SE* = 0.18, 95% CI [0.06, 0.75], a*RR* = 1.49, *P* = .022, *n* = 1850) and with the actigraphy/self-report composite (*B* = 0.91, *SE* = 0.23, 95% CI [0.46, 1.36], a*RR* = 2.48, *P* < .001, *n* = 645).

## Discussion

The current study reveals that having more sleep health problems may increase the risk of heart disease in middle adulthood. Results were consistent across our two models that used different sleep measures (i.e., self-report only and both actigraphy and self-report) and different samples. There were sex and race differences in each of sleep health and heart disease, however, the relationship between sleep health and heart disease did not differ by sex or race. Strengths of this study include the holistic assessment of sleep health using two different composites and replication of findings across different measures and samples. Findings show the utility of assessing multidimensional sleep health in predicting the risk of heart disease.

Our findings were similar to Brindle and colleagues^6^, who created a sleep health composite including self-reported measures of daytime alertness, quality, and timing, as well as actigraphy-based measures of regularity, efficiency, and duration. The current study extended this previous effort using a different approach. Our weighted composite approach is optimized for accurate prediction of heart disease and could be used in future studies as the regression weights were derived from a relatively large and representative sample of U.S. middle-aged adults. In our sensitivity analysis, we compared results with an unweighted sum composite, which assumes all sleep health dimensions contribute equally to the risk of heart disease. Results were generally consistent between these two approaches, with smaller effect sizes in the unweighted approach. The unweighted approach was similarly used in Brindle and colleagues^6^. yet our method is further distinguished by the use of a priori cutoffs established by the sleep expert panels^6,8,18,38,44^ that has high potential for replication across studies. For example, in our method using a priori cutoff for the sleep satisfaction/quality dimension, responses of “fairly bad” or “very bad” (vs. fairly good or very good) were identified as poor sleep quality, whereas Brindle and colleagues^6^ used ≥ 2.8 as an empirical cutoff that indicates poor sleep quality in their study sample. In other sleep health dimensions such as timing, efficiency, and duration, we used cutoffs that were used and validated by many studies to indicate poor sleep (e.g., < 85% for low sleep efficiency; see Table 3). Using their unweighted approach, Brindle and colleagues^6^ found that those with poorer sleep health were at 10% higher odds of cardiometabolic morbidity (*P* = .04). In our study, the risk of heart disease was 14% higher as a function of the unweighted self-report sleep health composite (*P* < .001). When we used our weighted approach, the detection of heart disease risk was much higher, such as 54% higher risk as a function of the self-report composite and 141% higher risk as a function of the actigraphy/self-report composite. Differences in the estimated risks may be attributed to differences in the samples as well as in the measures of sleep health and cardiovascular outcomes. Our supplemental results also showed that the significant effect of the self-report sleep health composite became non-significant when tested in the smaller actigraphy/self-report sample and the effect size was also much smaller than that of the actigraphy/self-report composite. Thus, when possible, using the actigraphy/self-report composite may increase the prediction of heart disease.

There were differences in the prevalence of heart disease and sleep health problems by sex and race, which was consistent with previous studies^28,30,31^. The link between sleep health and heart disease was not moderated by sex. The lack of moderation by sex may suggest that the relationship between multidimensional sleep health and heart disease is universal across men and women. There was a significant moderation by race in the association between actigraphy/self-report sleep health composite and heart disease, showing that the association was weaker for other racial minorities (including Native American, Asian, Native Hawaiian or Pacific Islander, and Hispanic) compared to non-Hispanic Whites. This may relate to a higher estimated risk of heart disease in other races regardless of their sleep health problems. We did not find a difference between non-Hispanic Blacks and Whites in the link between sleep health and heart disease; for both groups, having more sleep health problems significantly increased the risk of heart disease. However, note that effect sizes of sleep health composites varied by demographic sub-groups (Supplementary Table S1), suggesting a need to test potential moderation by these characteristics in a more diverse sample of adults. In particular, in this study, we lumped all other races together due to small % of the sub-groups (e.g., < 1% were Asian; see Table 4). Future studies could sample more individuals with other races to examine potential differences within this group. Examining potential differences in sleep health and heart disease by background characteristics is important because this helps identify at-risk groups.

Similar to previous research, we found that single sleep dimensions were associated with heart disease risk. However, the effect sizes of the individual sleep dimensions were weaker than those of sleep health composites that considered multiple sleep dimensions. Across the models, satisfaction dimension was consistently associated with the risk of heart disease, adjusting for other sleep dimensions. Previous studies found that insomnia^1,3,45^ and overall global sleep scores^31^ were related to increased risk of heart disease. Our findings further showed that the associations of these satisfaction measures with heart disease were independent of other sleep dimensions. Further, the associations were better understood when considering other sleep dimensions that may interact with sleep satisfaction. Note also that sleep duration (either based on self-report or actigraphy/self-report) was not significantly associated with heart disease. This is surprising given the reported associations of short or long sleep duration with multiple health outcomes^4,13,46^. This may be related to the characteristics of our sample, as most participants (78% in self-report sample; 56% in actigraphy/self-report sample) reported optimal sleep duration (≥ 6 & ≤ 8 h per night). It may also be that we examined independent association of sleep duration with heart disease (after controlling for other sleep dimensions), whereas other studies examined sleep duration as an isolated characteristic and did not adjust for potential shared variance with other sleep dimensions. Overall, this study clearly shows the importance of considering “co-exiting sleep health problems” in capturing the risk of heart disease. Our approach examining “how many” sleep health problems offers a comprehensive assessment of sleep health on one hand and the opportunity to better predict health risks on the other hand.

There are some limitations to consider. First, the current study is cross-sectional, which limits our ability to examine the effects of sleep health on heart disease risk over time. A future direction of this work is to analyze these associations longitudinally. Another limitation is the unbalanced sample sizes between our two models (6820 vs. 663), although it seems challenging to collect actigraphy sleep data in a larger sample. Further, the self-report sleep health composite could not capture sleep timing, as the larger survey did not include questions on bedtime and wake time. For other sleep dimensions, although we used the common sleep health framework^18^ to operationalize the two sleep health composites, specific sleep variables to capture each dimension may not be comparable between the composites and this is what we intended. Also, in the self-report sleep health composite, insomnia symptoms used to capture the satisfaction dimension may not coincide with the original conceptualization. More research is needed to examine where subjective insomnia symptoms fit into the sleep health dimensions theoretically and empirically. Another limitation is the lack of data available on sleep apnea, snoring, and bedtime use of electronic devices, all of which may influence one’s sleep health. Lastly, the majority of the MIDUS sample were White individuals and had higher education. Future studies could use more socioeconomically diverse samples, as socioeconomic status is closely linked to race^47^ and those with lower socioeconomic status are known to have more sleep complaints.

The current study shows the importance of considering “co-existing sleep health problems” within an individual to assess the risk of heart disease. Findings revealed having more sleep health problems may increase the risk of heart disease in middle adulthood. Results were consistent between two independent samples using different sleep health composites (using self-report only and both actigraphy and self-report). Despite known differences in the prevalence of sleep and heart disease by sex and race, the association between sleep health and the risk of heart disease did not generally differ by sex and race in our study. The findings highlight the importance and utility of assessing multidimensional sleep health in predicting the risk of heart disease and potentially other health outcomes.

## Supplementary Information

Supplementary Information.

## Data Availability

Data and documentation for all MIDUS projects are available to other researchers at the Inter-university Consortium for Political and Social Research (ICPSR). In addition to the publicly-available data at ICPSR, a MIDUS-Colectica Portal (midus.colectica.org) contains rich searchable metadata, links to helpful documentation, and the ability to download customized datasets.
