# Supplementary material for: Sleep health composites are associated with the risk of heart disease across sex and race
Source: Sci Rep. 2022 Feb 7;12:2023. doi: 10.1038/s41598-022-05203-0 (PMC8821698; doi:10.1038/s41598-022-05203-0)

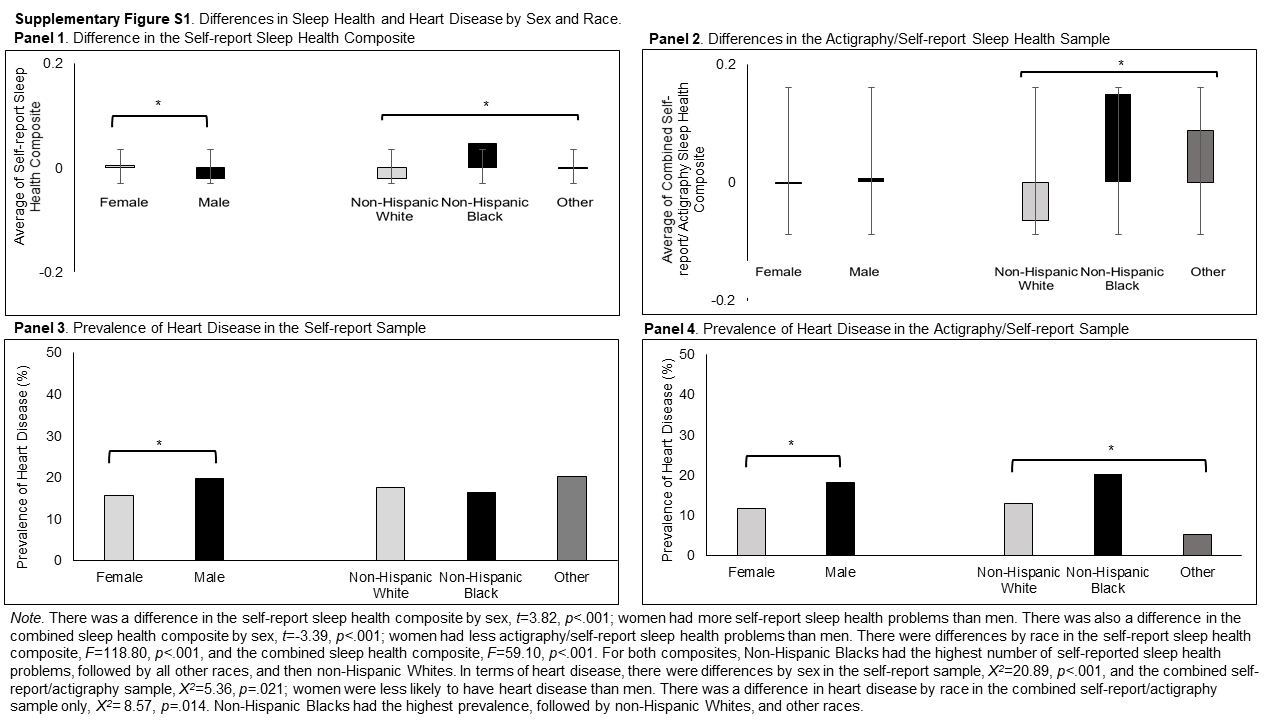


**Supplementary Table S1.** Results from models stratified by sex or by race predicting the relative risk of heart disease

|  | **Stratified by Sex** | |  | **Stratified by Race** | | |
| --- | --- | --- | --- | --- | --- | --- |
|  | **Females** | **Males** |  | **Non-Hispanic Whites** | **Non-Hispanic Blacks** | **All Other Races** |
| **Self-report Sleep Health Composite** | *B*=0.60, *SE*=0.13, 95% CI [0.36, 0.85], *aRR*=1.83, *P*<.001 | *B*=0.26, *SE*=0.11, 95% CI [0.03, 0.48], *aRR*=1.30, *P*=.024 |  | *B*=0.43, *SE*=0.10, 95% CI [0.23, 0.64], *aRR*=1.54, *P*<.001 | *B*=0.34, *SE*=0.21, 95% CI [-0.07, 0.75], *aRR*=1.41, *P*=.101 | *B*=0.45, *SE*=0.23, 95% CI [0.0006, 0.91], *aRR*=1.57, *P*=.049 |
| **Combined self-report/actigraphy Sleep Health Composite** | *B*=0.53, *SE*=0.45, 95% CI [-0.35, 1.40], *aRR*=1.69, *P*=.238 | *B*=1.05, *SE*=0.32, 95% CI [0.42, 1.68], *aRR*=2.85, *P*=.001 |  | *B*=1.44, *SE*=0.40, 95% CI [0.67, 2.22], *aRR*=4.24, *P*<.001 | *B*=0.86, *SE*=0.32, 95% CI [0.24, 1.48], *aRR*=2.37, *P*=.007 | Model did not converge due to small sample size |


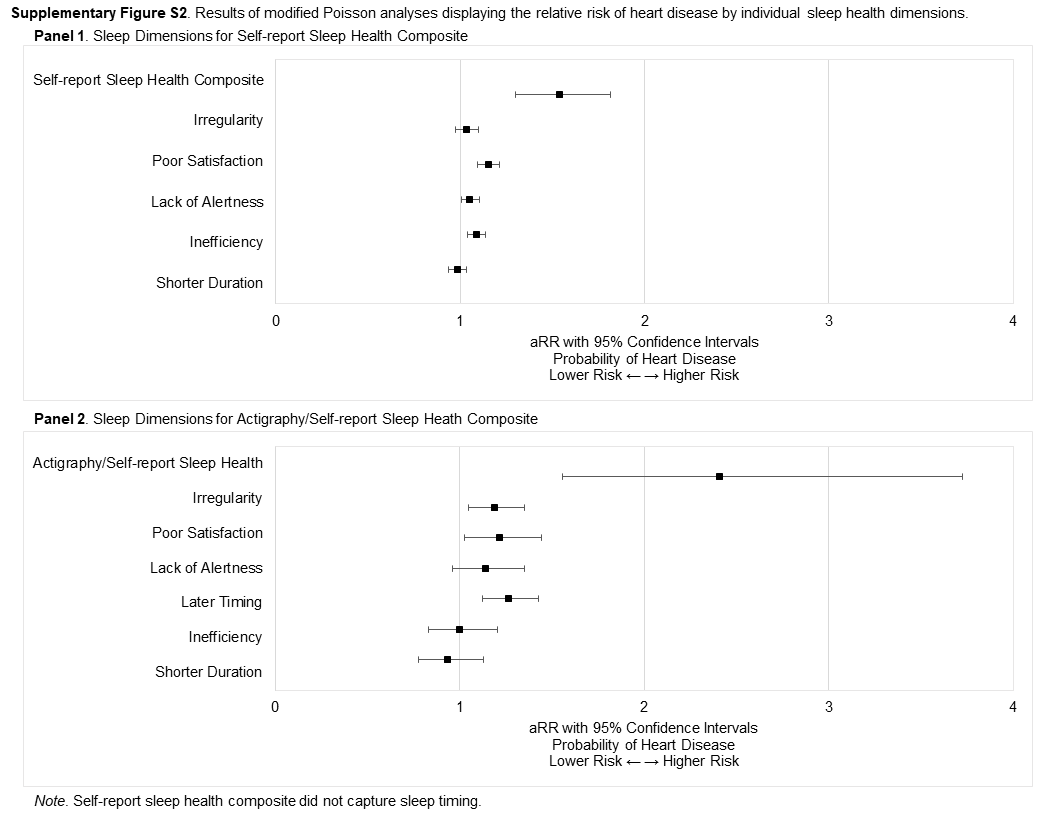

Supplement: Supplementary file 1 — Supplementary Information. [file 41598_2022_5203_MOESM1_ESM.docx]
